# Assessment of Glycemic Control in Patients With Diabetes in Northern Sudan Using Calculated HbA1c

**DOI:** 10.7759/cureus.33080

**Published:** 2022-12-29

**Authors:** Omer Abdel bagi, Ashraf Ewis

**Affiliations:** 1 Department of Pathology, Umm Al-Qura University, Al-Qunfudhah, SAU; 2 Department Public Health, Umm Al-Qura University, Al-Qunfudhah, SAU

**Keywords:** fasting blood glucose (fbg), diabetes mellitus, sudan, glycemic control, calculated hba1c

## Abstract

Background: Diabetes mellitus (DM) significantly burdens health services worldwide. As a simple and cost-effective method, the mathematical calculation of HbA1c is coming to be of value in areas with scarce resources. This study aimed to use calculated HbA1c to ascertain the prevalence of uncontrolled DM and correlate it with the risk factors for DM.

Methods: In the River Nile State of northern Sudan, a cross-sectional study was conducted in five leading cities from May to August 2021. Patients diagnosed and recorded as having type 2 or type 1 DM were included in this study. Enzymatic methods were used to assess fasting blood glucose (FBG). We used the mean of three FBG readings for three months to calculate HbA1c using the equation {HbA1c = (FBG mg/dl) x 0.03+2.6}, which was used to compute the estimated mediocre blood sugar over the course of three months.

Results: A total of 2047 diabetic patients from northern Sudan were studied for their DM control. Nearly two-thirds (65.2%) had uncontrolled DM. Of the patients studied, uncontrolled DM was significantly positively associated with older age, history of ischemic heart disease, and being a housewife. Multivariate regression analysis showed significant correlations between uncontrolled DM, an inactive lifestyle, and obesity.

Conclusion: The prevalence of uncontrolled DM among known patients with diabetic in northern Sudan is high (65.2%). The inactive lifestyles of housewives and freelance workers, having type 1 DM, and being hypertensive and obese are risk factors significantly associated with uncontrolled DM and its related complications.

## Introduction

Diabetes mellitus (DM) significantly burdens health services worldwide [1]. Around 642 million adults are expected to have diabetes in 2040 in low- and middle-income countries (LMICs) [2]. Countries moving from low to middle-income levels will show a high prevalence of diabetes due to lifestyle changes [3]. In Sudan, around 7.7% of adults are affected by diabetes, and the rate is increasing progressively [4,5]. A severe scarcity of healthcare workers exists in rural Sudan, and the problem is exacerbated by the inequitable allocation of health services [2]. Over 20 million Africans live with DM, which is alarming since the increasing prevalence of diabetes is a significant challenge to the healthcare system currently and in the future [6]. The overall prevalence of DM in north Sudan approaches 20 much higher than in 1998 (8.3%) [3]. Recent studies, in Suan showed that around 22.1% of adults were affected by DM in 2019, and the prevalence is expected to be 23.5% and 24.2% in 2030 and 2045, respectively [7-9]. Checking glycemic control in patients with diabetes is of utmost importance in assessing the prognosis and modifying the risk factors [10]. Identifying the risk factors by raising awareness and educating the patients can help prevent DM-related complications [11,12]. In Sudan, the DM complication rate is estimated to be 5.44%, especially in older patients with long-duration diabetes and concomitant comorbidities. Diabetes is one of the most critical major modifiable risk factors of macro and microvascular disease, which may end up in blindness, renal failure, acute coronary syndrome (ACS), and ischemic heart disease (IHD) [13,14].

Additionally, around 10 to 25% of patients with diabetes are likely to develop diabetic foot (DF) at some stage of their lives [15,16]. Estimating the fasting blood glucose (FBG) level and glycated hemoglobin A1c (HbA1c) is the best tool for both monitoring and diagnosis of DM [10]. HbA1c is used to assess average plasma glucose level in the previous 3-4 months (life span of RBCs). HbA1c is proven to be affected by RBC’s life span (120 days), and the assessment includes all RBCs ages from oldest to youngest. Some studies revealed that if we do continuous blood glucose monitoring, the contribution of the last thirty days will be more than 50%, while the contribution of the blood glucose levels in the early 90 days contribute by only 10%, which explains the marked variation of HbA1c with a quick variation of blood glucose [17,18]. While doing an HbA1c assessment, in case of inappropriate results correlated with clinical findings, the pathologist and technologist must search for hemoglobin variants and use the appropriate method of HbA1c assessment since some interfere with enzymatic processes. In contrast, others interfere with immunoassay [18]. Being simple and cost-effective, the mathematical calculation of HbA1c is becoming of value in areas with insufficient resources [19]. In areas with low resources, such as our setting in northern Sudan, the availability of HbA1c assessment is a big issue; hence the calculated HbA1c is a reasonable solution to assess glycemic control [19]. This study aims to use the calculated HbA1c to assess the prevalence of uncontrolled DM and correlate it with the risk factors of DM.

## Materials and methods

Study area and design: In the River Nile State in northern Sudan, this cross-sectional study was conducted in the five main cities of Ad-dammar, Atbara, Shandi, Abu Hamed, and Berber from May to August 2021.

Study population: Patients diagnosed and recorded with type 2 or type 1 DM for at least a year in the five cities were included in this study. Patients aged less than 18 years, pregnant, elderly with poor mental competence, and morbidly ill patients were excluded from the study. Elsheikh Abdullah Elbadry University granted ethics approval for this study (2021/2). All procedures were carried out in line with the Helsinki Declaration. All participants provided informed consent.

Methods: Enzymatic methods were used to assess FBG using a calibrated, fully automated chemical analyzer (Biosystems analyzer A 15 Barcelona- Spain). If FBG is less than 130 mg/dl (7.2 ml mole per liter), it indicates reasonable glycemic control. But when FBG equals 130 mg/dl or more, the glycemic control is poor. The American Diabetes Association (ADA) claims that blood sugar control is defined as HbA1c less than 7% for excellent control and more than 7% for poor control [19]. The literature revealed a clear linkage between the FBG and HbA1c; we use the mean of three FBG readings for three months to calculate HbA1c using the equation {HbA1c = (FBG mg/dl) x 0.03+2.6} which is used to compute the estimated mediocre blood sugar over the course of three months. A qualified nurse measured blood pressure, and hypertension was defined as a patient who was already taking antihypertensive medication or whose blood pressure after three separate measurements was > 140/90 mmHg. Participants weighed themselves on a Seca scale while wearing no shoes. A standard height board was used to measure their heights. Body mass index (BMI) is computed by dividing weight in kilograms by height in square meters, normal (< 25/kg/m^2^), overweight (25-29.99 kg/m^2^), and obese (greater than 30 kg/m^2^).

Data collection: A well-prepared questionnaire was utilized to collect sociodemographic data, family history, diabetes duration, residency, and comorbidities.

Statistical analysis: Data analysis was performed using the statistical package for social sciences (SPSS) for Windows was used to analyze the data (version 25.0). The proportion of patients with controlled and uncontrolled DM was compared using the Chi-squared test. Odd’s ratio (OR) and the adjusted OR (AOR) were calculated to assess the risk factors. The independent variable was incorporated into the multivariate logistic regression analysis model. P-values of less than 0.05 were considered significant.

## Results

A total of 2047 patients with diabetes from northern Sudan were studied for their DM control. Nearly two-thirds (65.2%) of the patients had uncontrolled DM. The number of females (54.1%) was slightly higher than males, with the mean age of the studied patients being 56.45 (±12.43) years. Most (70%) of the patients were of type 2 DM, with a mean disease duration of 6.76 years. Almost half of the patients with diabetes had a positive family history of DM. The dominant occupation was a housewife and the major contribution of participants was from Atbara (45%) (Table 1).

**Table 1:** Sociodemographic data and clinical characteristics of the studied adult diabetic patients from Sudan

Variable		Frequency	Percent
Sex	Female	1110	54.2%
	Male	937	45.8%
Occupation	Housewife	880	43.0%
	Employee	581	28.4%
	Student	67	3.3%
	Freelancer	519	25.4%
	Housewife	880	43.0%
City	Barbar	464	22.7%
	Abu Hamad	344	16.8%
	Atbara	555	27.1%
	Al Damar	375	18.3%
	Shandi	309	15.1%
Diabetes type	Type 1	612	29.9%
	Type II	1435	70.1%
Hypertension	Absent	1072	52.4%
	Present	975	47.6%
Duration	Less than 5 years	697	34.0%
	5 years and above	1350	66.0%
Fasting blood glucose	less than 130 mg/dl	713	34.8%
	130 mg/dl and above	1334	65.2%
Body mass index	Underweight	23	1.1%
	Normal	483	23.6%
	Overweight	760	37.1%
	Obese 1	448	21.9%
	Obese 2	333	16.3%

In the patients studied, uncontrolled DM was significantly associated with age, history of IHD, being a housewife, residency in Barbar and Atbara, obesity, having type 2 DM, incantatory uncontrolled diabetes not affected by family history or those who are less than 50 years (Table 2).

**Table 2:** Comparison between the controlled and uncontrolled diabetes mellitus among the studied adult diabetic patients from Sudan

		FBG < 130 mg/dl	FBG ≥ 130 mg/dl	p-value
		(N=713)	(N=1334)	
Age (y)	Mean ±SD (Range)	55.7±10.1 (22-83)	56.9±13.5 (15-84)	0.038*
Age ≥30	≥ 30 y	709 (99.4%)	1271 (95.3%)	<0.001*
Age ≥40	≥ 40 y	665 (93.3%)	1219 (91.4%)	0.133
Age ≥50	≥ 50 y	599 (84%)	1030 (77.2%)	<0.001*
Sex	Female	371 (52%)	739 (55.4%)	0.146
	Male	342 (48%)	595 (44.6%)	
Occupation	Housewife	291 (40.8%)	589 (44.2%)	<0.001*
	Employee	261 (36.6%)	320 (24%) *	
	Student	4 (0.6%)	63 (4.7%) *	
	Freelancer	157 (22%)	362 (27.1%) *	
City	Barbar	167 (23.4%)	297 (22.3%)	<0.001*
	Abu Hamad	137 (19.2%)	207 (15.5%) *	
	Atbara	216 (30.3%)	339 (25.4%) *	
	Al Damar	130 (18.2%)	245 (18.4%)	
	Shandi	63 (8.8%)	246 (18.4%) *	
Diabetes type	Type 1	137 (19.2%)	475 (35.6%)	<0.001*
	Type II	576 (80.8%)	859 (64.4%)	
Disease duration	Mean±SD (Range)	7.1±5.1 (1-20)	6.5±4.4 (1-20)	0.006*
	Less than 5 years	224 (31.4%)	473 (35.5%)	0.066
	5 years and above	489 (68.6%)	861 (64.5%)	
Diabetic foot	No	637 (89.3%)	1150 (86.2%)	0.042*
	Yes	76 (10.7%)	184 (13.8%)	
Hypertension	Absent	431 (60.4%)	641 (48.1%)	<0.001*
	Present	282 (39.6%)	693 (51.9%)	
Family history	No	398 (55.8%)	704 (52.8%)	0.188
	Yes	315 (44.2%)	630 (47.2%)	
Ischemic heart disease	No	690 (96.8%)	1320 (99%)	<0.001*
	Yes	23 (3.2%)	14 (1%)	
Body mass index	Mean ±SD (Range)	28.95±5.77 (15.8-45.6)	28.99±5.77 (14.4-45.2)	0.879
	Underweight	6 (0.8%)	17 (1.3%)	<0.001*
	Normal	158 (22.2%)	325 (24.4%)	
	Overweight	309 (43.3%)	451 (33.8%) *	
	Obese 1	123 (17.3%)	325 (24.4%) *	
	Obese 2	117 (16.4%)	216 (16.2%)	
Obese	BMI ≥ 30	240 (33.7%)	541 (40.6%)	0.002*
	BMI < 30	473 (66.3%)	793 (59.4%)	

A multivariate regression analysis showed that there were significant correlations between uncontrolled DM and having an occupation as an employee (AOR = 0.46 (0.37-0.59), p <0.001), type 1 DM (AOR = 2.52 (1.98-3.2), p <0.001), hypertension (AOR 1.51 (1.24-1.84) p <0.001), and obesity (AOR 1.36 (1.11-1.67) p<0.003) (Table 3).

**Table 3:** Univariate and multivariate analysis of the factors associated with uncontrolled diabetes mellitus among the studied adult diabetic patients from Sudan

	Univariate analysis		Multivariate analysis	
	Crude OR (95% CI)	p-value	AOR (95% CI)	p-value
Age (≥ 30 y)	0.11 (0.04-0.31)	<0.001*		
Sex (male)	1.15 (0.95-1.37)	0.146		
Occupation				
Housewife	1 (reference)			
Employee	0.61 (0.49-0.75)	<0.001*	0.46 (0.37-0.59)	<0.001*
Student	7.78 (2.81-21.59)	<0.001*	3.8 (1.33-10.83)	0.013*
Freelancer	1.14 (0.9-1.44)	0.275	1.04 (0.82-1.33)	0.741
Diabetes type (Type 1)	2.33 (1.87-2.89)	<0.001*	2.52 (1.98-3.2)	<0.001*
Disease duration (y)	0.97 (0.96-0.99)	0.006*	0.97 (0.95-0.99)	0.002*
Diabetic septic foot	1.34 (1.01-1.78)	0.043*		
Hypertension	1.65 (1.37-1.99)	<0.001*	1.51 (1.24-1.84)	<0.001*
Family history	1.13 (0.94-1.36)	0.188		
Body mass index	1 (0.99-1.02)	0.879		
Obese	1.35 (1.11-1.63)	0.002*	1.36 (1.11-1.67)	0.003*

## Discussion

In this study, we used calculated HbA1c to assess glycemic control, drawing on previous valuable and reliable research. The relationship between HbA1c and FBG is evident, especially in diabetic patients [20]. Ghazanfari et al. (2010) studied the relationship between HbA1c and FBG and concluded that FBG is an accurate predictor of HbA1c. Moreover, in a comparative study using high-pressure liquid chromatography (HPLC) and cost-effective calculated HbA1c, Dayanand et al. (2012) found a significant association between HPLC measured and the mathematically calculated HbA1c [21-23]. In many sub-Saharan countries, including our study area, the explanation for poor glycemic control is mainly the cost of drugs and investigations; some good and effective medicines are doubtlessly beyond the reach of most patients, and some studies, in addition to a lack of awareness of long-term complications of DM [22]. Many conditions, whether pathological or physiological, can affect the results of HbA1c and must be considered when interpreting the results. These conditions include hemoglobinopathies, uremia, pregnancy, hemodialysis, alcohol intake, and aspirin administration [23]. There is also a problem with lifelong conditions, such as hemoglobinopathies; it is worth mentioning that sickle cell anemia prevalence in Sudan ranges from 2.5 to 30% [22]. In settings like that of our current study area and similar areas in other developing countries, besides these universal problems of HbA1c investigation, there are many local obstacles, such as the availability of machines, availability and stability of electricity, availability of good quality machines, availability of calibrating and standard reagents, and the cost and charges for the relatively expensive investigations. In this study, the prevalence of uncontrolled DM was found in 65.2% of the patients with diabetes studied. Similar findings were seen in eastern Sudan, Uganda, and Saudi Arabia, where uncontrolled DM accounted for 71.9% [24], 68% [22], and 59.3% [25], respectively, indicating that using calculated HbA1c gave almost similar results to studies done by stander procedure to assess long-term glycemic control. Studies from Sudan, Ethiopia, and western Africa revealed a very high prevalence of uncontrolled DM in patients with type 2 diabetes, with scores of 85% [26], 86%, [27], and 83.8% [28], respectively. The difference is probably due to variations in the investigation methods, the number of patients studied besides patient awareness and the lack of continuous health education. Lower scores for the prevalence of uncontrolled DM were reported in studies from Nigeria (40%) [29] and Kenya (27.1%) [30]. Our study showed a strong correlation between age, history of IHD, and type 2 diabetes; this finding agrees with [31-33]. This can be explained by the strong correlation between DM and vascular disease, especially in prolonged diseases, and because type 2 diabetes is known to be a disease of older age. Being a housewife showed a strong correlation with uncontrolled DM, which is in line with the findings of other previous studies, such as a study in Nepal [34]. This can be explained by inactive lifestyle, dietary habits, and the fact that most women in this part of Sudan prefer not to work outside their houses because of tradition. There was also a relationship between a patient’s residence in Barbar and Atbara and poor glycemic control, most probably due to the semi-sedentary life in these cities. Our study showed type 1 DM to be an independent factor for poor glycemic control; this agreed with a study from Dar Es Salaam [35]. This can be explained by the fact that type 1 mainly affects children, and it is challenging to control dietary habits and proper use and storage of insulin. The current study also showed a positive correlation between hypertension risk (OR = 1.15) and uncontrolled DM, a finding similar to the results reported in Nepal [36]. Obese patients in this study showed a high risk of developing uncontrolled DM (1.36-fold), a finding like those reported by many studies from different African countries [2,6,8,10,13]. The limitation of this study includes some issues that were not addressed, such as physical activity, lipid profile, smoking, and dietary habits.

## Conclusions

The prevalence of uncontrolled DM among known diabetic patients in northern Sudan is high (65.2%). The inactive lifestyles of housewives and freelance workers, having type 1 DM, and being hypertensive and obese are risk factors significantly associated with uncontrolled DM and its related complications. We recommend that calculated HbA1c be used in areas with difficulties conducting HbA1c measurements.
